# Colovesical Fistulae: The Varying Aetiologies

**DOI:** 10.7759/cureus.20025

**Published:** 2021-11-29

**Authors:** Mansoor Zafar, Sara Lee, Serena Tieger, William Sacre, Mark Whitehead

**Affiliations:** 1 Gastroenterology and Hepatology, and General Internal Medicine, Conquest Hospital, East Sussex Healthcare NHS Trust, St. Leonards-on-Sea, GBR; 2 General Internal Medicine, Conquest Hospital, East Sussex Healthcare NHS Trust, St. Leonards-on-Sea, GBR; 3 Internal Medicine, Conquest Hospital, East Sussex Healthcare NHS Trust, St. Leonards-on-Sea, GBR; 4 Radiology, Conquest Hospital, East Sussex Healthcare NHS Trust, St. Leonards-on-Sea, GBR; 5 Gastroenterology, Conquest Hospital, East Sussex Healthcare NHS Trust, St. Leonards-on-Sea, GBR

**Keywords:** multi-disciplinary team approach, ct-abdomen & pelvis (portal venous phase), mri fast relaxation fast spin echo sequence), contrast-enhanced ct-abdomen & pelvis, colo vesical fistulae

## Abstract

The most common presenting symptoms of colovesical fistulae (CVF) are pneumaturia and fecaluria. The most important aspect remains not only to investigate the aetiology, and the degree of both severity and complexity, but also the subsequent influence of this on overall management. In a younger population, management usually consists of curative surgery. However, this may not be possible in older patients where surgical candidacy is a genuine concern and a clinical challenge arises relating to pursuing a conservative strategy. We attempted to briefly outline how two patients were managed with a similar non-surgical approach due to frailty. These cases attempt to highlight the importance of multi-disciplinary specialty input, with a view to optimising patient care.

## Introduction

A colovesical fistula (CVF) is defined as an abnormal connection between the colon and urinary bladder [1]. The precise incidence of CVF is unknown: it is estimated that they account for 1 in every 3000 surgical hospital admissions [1]. In patients with diverticular disease, 2-18% were found to have CVF [2-5].

The male-to-female ratio of CVF is approximately 2:1 to 3:1 [6-10]. It has been hypothesised that females are protected from developing CVF due to the presence of the uterus and broad ligaments acting as a barrier between the sigmoid colon and the bladder. This theory is supported by the fact that a high percentage of females with CVF (>50% in some series) have had a previous hysterectomy [11].

We present a case series report of two female patients, with different aetiologies contributing to the development of colovesical fistulae, along with the sequelae.

## Case presentation

Case series report

Case 1

An 83-year-old female was admitted with dysuria, pneumaturia, and malaise. Past medical history included diverticulosis, hypertension, gout, Barrett’s oesophagus, recurrent urinary tract infections (UTIs), stable non-Hodgkin's lymphoma, and total abdominal hysterectomy (1983). On examination, baseline observations were stable, and mild suprapubic tenderness was noted. A bedside urine test was positive for nitrites and leukocytes; the urine culture identified *Escherichia coli* with extended-spectrum beta-lactamase (ESBL) positivity. Admission blood tests showed a mildly raised C-reactive protein (CRP) 32 [5], with a normal white cell count (WCC) of 6.08 × 109/L [4-11]. Initial management included intravenous fluid resuscitation and antibiotics.

**Figure 1:**
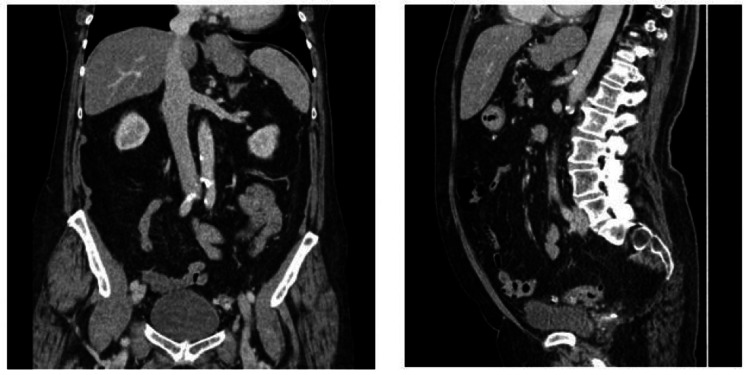
Contrast-enhanced CT-abdomen and pelvis (portal venous phase): coronal and sagittal views. There are no indirect signs or obvious adherence of sigmoid colon to the urinary bladder. However, it is not possible to definitively exclude a fistula on CT and, given the clinical details, an MRI scan may be useful to further evaluate these tissues in more detail.

**Figure 2:**
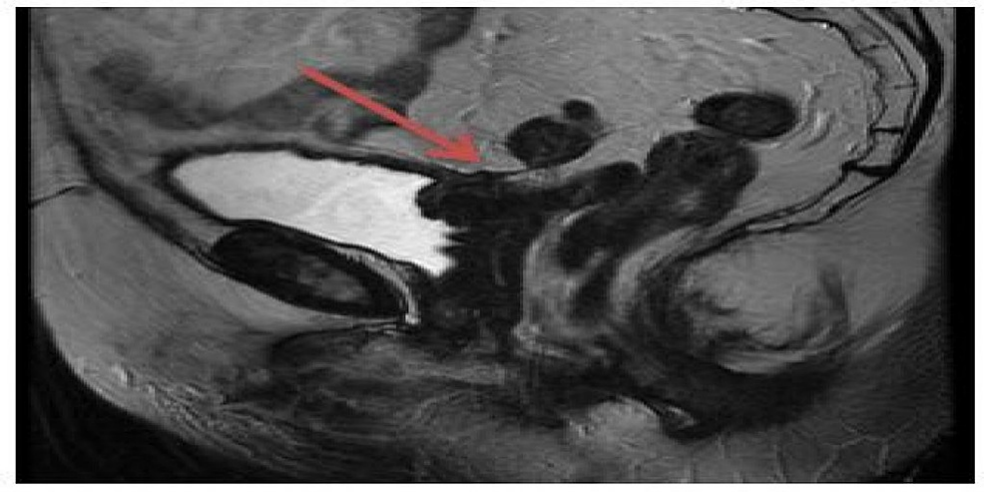
MRI-pelvis: sagittal view (T2 fast relaxation fast spin-echo sequence). Linear area of soft tissue tethering the distal sigmoid colon to a thickened area of the posterosuperior urinary bladder wall. These features likely represent a colovesical fistula.

Given the history of recurrent UTIs and recent pneumaturia, a CT-abdomen and pelvis was performed to exclude a colovesical fistula (Figure 1). Subsequent to this, magnetic resonance imaging (MRI) of the pelvis was undertaken, which demonstrated findings consistent with a colovesical fistula (Figure 2). The case was discussed in a multi-disciplinary meeting with input from clinical specialties including gastroenterology, general surgery, and radiology. Non-surgical management of the CVF with oral antibiotics was the agreed outcome and the patient was discharged with a course of nitrofurantoin.

Case 2

**Figure 3:**
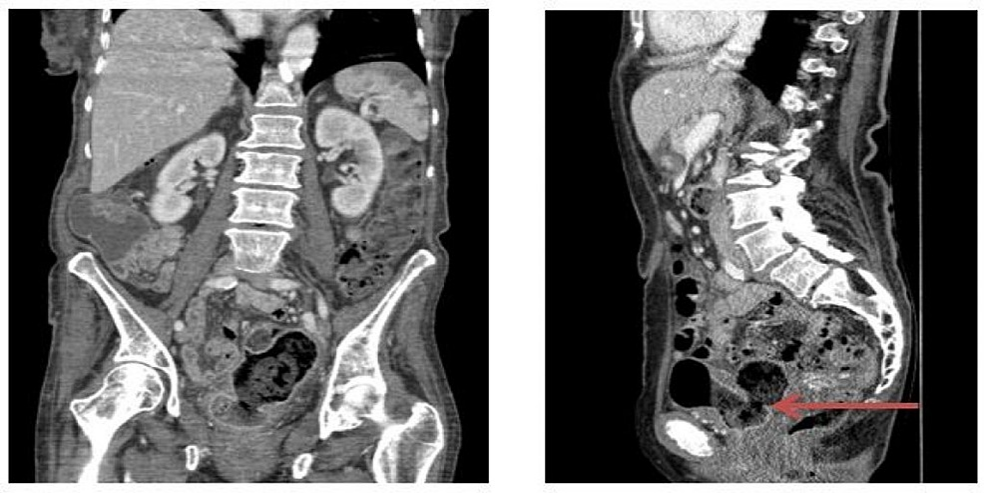
Contrast-enhanced CT-abdomen and pelvis (portal venous phase): coronal and sagittal views. The wide-necked connection between the proximal sigmoid and the bladder with extensive faecal residue filling the bladder, consistent with a colovesical fistula.

An 88-year-old female presented to the emergency department with acute confusion. Past medical history included a recent diagnosis of type 1 diabetes mellitus, severe frailty, multiple falls, sigmoid diverticulosis. Initial baseline observations were unremarkable. Admission blood tests were essentially unremarkable. A bedside urine test was positive for leukocytes and nitrite; the subsequent culture revealed >105/cm^3^ heavy mixed growth. Initial management consisted of intravenous fluids and antibiotics. A CT-head excluded an acute intracranial event. Due to the recent diagnosis of type 1 diabetes mellitus in an older patient, a CT-abdomen and pelvis was performed to exclude a pancreatic malignancy. Whilst no concerning pancreatic pathology was identified, a large colovesical fistula was incidentally identified (Figure 3).

The case was discussed in a multi-disciplinary meeting with input from clinical specialties including gastroenterology, general surgery, urology, and radiology. A decision was made in light of her severe frailty for non-surgical management. Following advice from microbiology, alternating prophylactic antibiotic regimes were commenced, rotating every three months. Periodic follow-up in the surgical outpatient clinic was also arranged and the patient was discharged.

## Discussion

The first description of a colovesical fistula has been reported to be attributed to Rufus of Ephesus in AD 200 [12]; however, it was in 1888 that Cripps produced his classic monograph on the subject [11].

A number of causes have been reported to be the aetiology of colovesical fistulae, with the most notorious being a diverticular disease, as well as a sequel to inflammatory bowel disease, more specifically Crohn’s disease [13,14]. Holmes et al. have reported incidence with colorectal, gynaecological, and urological malignancies [14,15]. Subsequent to a foreign body in the gastrointestinal tract [16], laparoscopic and open inguinal hernia repairs [17] and benign gastrointestinal and urological diseases [18,19] have also been reported. Kiani et al. conducted a comprehensive study at Guy's and St. Thomas' NHS Trust of 55 patients with a diagnosis of CVF. They concluded that although having a similar clinical presentation, colovesical fistulae of various aetiologies differ significantly in management and outcome [19].

We attempted to highlight the case series report of two patients. One case had the usual sequel including a background history of abdominal hysterectomy. The second case presented with no appropriate cause or iatrogenic background, presenting as a clinical challenge. Both patients were managed in a similar way following a conservative approach due to frailty and patient autonomy. This emphasizes that patients can be managed conservatively in cases where surgical intervention may not be appropriate.

## Conclusions

Colovesical fistulae can occur with or without previous history of the bowel, bladder, or gynaecological procedures. Whilst surgical input is justified in the younger population, management should be decided on a case-by-case basis, with a non-surgical approach being suitable among a frailer populace. Utilisation of imaging together with a multi-disciplinary specialty approach within secondary care permits a patient-centered approach to CVF management.
